# Mapping PTSD symptoms to brain networks: a machine learning study

**DOI:** 10.1038/s41398-020-00879-2

**Published:** 2020-06-18

**Authors:** Amin Zandvakili, Jennifer Barredo, Hannah R. Swearingen, Emily M. Aiken, Yosef A. Berlow, Benjamin D. Greenberg, Linda L. Carpenter, Noah S. Philip

**Affiliations:** 1 Department of Psychiatry and Human Behavior, Alpert Medical School of Brown University, Providence, RI 02906 USA; 2 VA RR&D Center for Neurorestoration and Neurotechnology, Providence VA Medical Center, Providence, RI 02908 USA

**Keywords:** Diagnostic markers, Psychiatric disorders, Diagnostic markers, Psychiatric disorders

## Abstract

Posttraumatic Stress Disorder (PTSD) is a prevalent and debilitating condition with complex and variable presentation. While PTSD symptom domains (intrusion, avoidance, cognition/mood, and arousal/reactivity) correlate highly, the relative importance of these symptom subsets often differs across patients. In this study, we used machine learning to derive how PTSD symptom subsets differ based upon brain functional connectivity. We acquired resting-state magnetic resonance imaging in a sample (*N* = 50) of PTSD patients and characterized clinical features using the PTSD Checklist for DSM-5 (PCL-5). We compared connectivity among 100 cortical and subcortical regions within the default mode, salience, executive, and affective networks. We then used principal component analysis and least-angle regression (LARS) to identify relationships between symptom domain severity and brain networks. We found connectivity predicted PTSD symptom profiles. The goodness of fit (*R*^2^) for total PCL-5 score was 0.29 and the *R*^2^ for intrusion, avoidance, cognition/mood, and arousal/reactivity symptoms was 0.33, 0.23, −0.01, and 0.06, respectively. The model performed significantly better than chance in predicting total PCL-5 score (*p* = 0.030) as well as intrusion and avoidance scores (*p* = 0.002 and *p* = 0.034). It was not able to predict cognition and arousal scores (*p* = 0.412 and *p* = 0.164). While this work requires replication, these findings demonstrate that this computational approach can directly link PTSD symptom domains with neural network connectivity patterns. This line of research provides an important step toward data-driven diagnostic assessments in PTSD, and the use of computational methods to identify individual patterns of network pathology that can be leveraged toward individualized treatment.

## Introduction

Posttraumatic Stress Disorder (PTSD) is a highly prevalent and chronic psychiatric disorder, characterized by trauma exposure, followed by intrusive thoughts/recollections, avoidance of related stimuli, hyperarousal, and mood and cognitive impairment^1,2^. In the USA, lifetime prevalence is estimated at 7%, with higher prevalence in military Veterans^1–3^. Current evidence-based treatments, including psychopharmacology and psychotherapy, are often inadequately effective^3^. In addition to suffering due to the symptoms themselves, PTSD is also associated with poor functioning and disability, general medical illness, and poor quality of life^4–6^.

Though the consequences of PTSD are well established, its presentation is heterogeneous. Diagnosis is often difficult, as PTSD is highly comorbid with other psychiatric illnesses^7,8^ and individuals may possess a myriad of symptoms. Different diagnostic and nosological models attempt to identify PTSD symptoms—this is evident in the DSM-5^2^ criteria for PTSD, which groups symptoms in four domains: intrusion (criterion B); avoidance (criterion C); cognition and mood (criterion D); and Arousal and reactivity (criterion E). Symptom groupings are based upon their frequent co-occurrence in observational studies, or by data-driven approaches such as factor analysis^9,10^. Finding a biological biomarker can aid clinical diagnosis and remove biases and uncertainties that can occur during the course of clinical practice. Finding such a unified biomarker for PTSD has proven difficult, likely given the diversity and heterogeneity of presentation (e.g.,^11^), indicating that different biological subtypes exist within the clinical symptom profile.

One novel approach to developing objective markers of PTSD is through the use of functional neuroimaging to examine neural circuits (reviewed in^12,13^) to identify biological correlates of symptom domains. The brain is organized into discrete neural networks (e.g.,^14,15^), and recent work has described multiple alterations in these networks in PTSD. For example, PTSD is associated with deficits within the frontoparietal network (FPN), increased salience network (SN) connectivity and disruptions in default mode network (DMN) connectivity^16^. Studies that have investigated the relationship of PTSD symptom domains to deficits in brain networks have typically relied on exposure and imagery during functional imaging to elucidate the association of specific brain networks with different PTSD symptoms^17–19^. Other imaging studies have used topological approaches to characterize how PTSD impacts neural networks^20,21^, and recent work suggests the utility of using machine learning approaches to predict and potentially identify those at risk for PTSD^22^. This area of inquiry has already significantly advanced the field in psychosis research and substance use, where machine learning can now identify patients using brain-based pathology^23^ and individualized treatment response^24,25^.

Currently, it is unknown if a combined neuroimaging and machine learning approach will provide similar links between pathological neural circuits and individualized symptom profiles in PTSD. Doing so would represent a first step toward characterizing the neural basis of heterogeneity in PTSD. If successful, potential objective markers of domain-level pathology may serve as targets for future circuit-based, personalized interventions. Though transcranial magnetic stimulation can reduce PTSD symptoms [e.g.,^3,26,27^, reviewed in^28^], response to stimulation is reduced in those with more severe symptoms in some domains^29^. Therefore, there is a need to identify novel targets or circuits to engage to maximize treatment efficacy (e.g.,^30^). Here, we applied a machine learning approach to identify brain functional networks as they relate to PTSD symptom domains, derived from patient-reported scales, hypothesizing that we would be able to identify novel relationships between data-driven connectivity patterns and individual PTSD symptom domains.

## Patients and methods

### Overview

Following informed consent, magnetic resonance imaging data were acquired from 50 participants on a 3T MRI scanner at Brown University (see Supplementary methods for further information on neuroimaging acquisition, preprocessing, and quality control). The Providence VA Medical Center and Butler Hospital Institutional Review Boards approved these studies, and identical procedures were used at both sites. The data used for the analyses presented here were recorded as part of pretreatment (i.e., baseline) scanning in three previous studies from our group^29,31,32^.

### Participants and assessments

Participants (*N* = 50) were 48.84 ± 11.78 years of age, and 38% (*n* = 13) were women. All participants met DSM-5 criteria for PTSD. Self-reported PTSD symptoms were measured using the PTSD Checklist for DSM-5 PTSD (PCL-5;^33^). The PCL-5 is a 20-question scale and provides a score between 0–80, correlated with PTSD severity. Furthermore, the PCL-5 score can be divided into four subscales, corresponding to PTSD symptom domains described above. The majority of participants also had major depression, which would be expected due to the high comorbidity between these two disorders^34^, and the vast majority were on concurrent pharmacotherapy. If applicable, all participants were receiving stable treatment (e.g., medications and psychotherapy) for at least 6 weeks before neuroimaging. Inclusion/exclusion criteria and full participant information is included in the Supplementary materials (see Supplementary Table 1).

### Region-of-interest selection

We selected 100 cortical and subcortical regions-of-interest (ROIs) for functional connectivity analyses. ROIs were located in functional networks implicated in PTSD by prior studies (Table 1; Supplementary Fig. 1). ROIs were inclusive of the DMN, SN, FPN and affective network (AN). ROIs were defined using the Human Connectome Project Multimodal Atlas^35^; amygdala and hippocampus were based on the probabilistic atlas of Mazziotta et al.^36^, while striatal ROIs are from the seven-network functional parcellation of Choi et al.^37^ The selection of areas limited the dimensionality and make the regression computationally tractable (see ‘Discussion’) and was made a priori to all analyses.

**Table 1:** List of included ROIs.

Network	Anatomical group	ROI
Subcortical		
	Medial temporal lobe	Amygdala (CM)
		Amygdala (BL)
		Ant hippocampus
		Mid hippocampus
		Pos hippocampus
	Basal ganglia and thalamus	Striatum (FPN)
		Striatum (DMN)
		Thalamus (PFC)
Affective		
	VMPFC	10r, 10v
	Subgenual	25, s32
	Orbital	11l, 13l
		OFC, pOFC
Default		
	DLPFC	9p
	MPFC	10pp, a10p, p10p
		10d, 9m
	Orbital	47s, 47m, a47r
	Ant cingulate and paracingulate	a24, p24, p32
	PCC	v23ab
FPN		
	DLPFC	9–46d, a9-46v, p9-46v
		46
	Inf frontal cortex	47l, p47r
	VLPFC	44, 45
		IFSa, IFSp
	Mid cingulate and paracingulate	23c, d23ab
SN		
	DLPFC	9a
	Ant to Mid cingulate	a24pr, p24pr
	Ant paracingulate	d32, a32pr, p32pr
	Mid paracingulate	23d, 24dd, 24dv

For ROIs based on the Human Connectome Project Multimodal Atlas, the prefix ‘a’ or ‘p’ usually denotes an anterior or posterior subregion of regions typically found in unimodal atlases (e.g., Brodmann’s areas). The same is true for ‘d,’ ‘v,’ ‘r,’ ‘m,’ ‘l,’ which stand for ‘dorsal,’ ‘ventral,’ ‘rostral,’ ‘medial,’ and ‘lateral’, respectively. ‘CM’ and ‘BL’ in the subcortical ROIs refer to the centromedial and basolateral divisions of the amygdala, respectively.

*DMN* default mode network, *PFC* prefrontal cortex, *MPFC* medial prefrontal cortex, *FPN* frontoparietal network, *SN* salience network, *VMPFC* ventromedial prefrontal cortex, OFC orbitofrontal cortex, *pOFC* posterior orbitofrontal cortex, *MTL* medial temporal lobe, *CM* centromedial, *BL* basolateral.

### Subject-level ROI-to-ROI functional connectivity analysis

For each subject, functional MRI time courses were extracted from preprocessed functional data for each ROI. Time courses were entered into a cross-correlation matrix and the resulting bivariate Pearson’s correlations, calculated in 4950 unique ROI pairs. We used the absolute value of Pearson correlation as a measure of functional connectivity.

### Machine learning

We first used principal component analysis to reduce the dimensionality of the functional connectivity matrix. We selected the first principal components that cumulatively represented 90% of the variance in the functional connectivity matrix, compressing the 4950 dimensions of the connectivity matrix to 39 (i.e., the first 39 components represented 90% of data variance, see Supplementary Fig. 2). We then used least-angle regression (LARS)^38^ on this dimensionality-reduced dataset to predict each of the four PCL-5 subscales: criterion (B) intrusion, (C) avoidance, (D) cognition/mood, and (E) arousal/reactivity. The LARS algorithm provides a parsimonious regression model for efficient prediction of a response variable, particularly when the number of predictor variables is large. The ability of the regression models to predict symptoms was tested, utilizing full iterative leave-one-out cross-validation to evaluate the performance and calculating coefficient of determination (*R*^2^) using the below formula:

$$R^2 \equiv 1 - \frac{{\mathrm{SS}_{\mathrm{res}}}}{{\mathrm{SS}}_{\mathrm{tot}}},$$

where SS_res_ was the sum of squares of residuals and SS_tot_ was the total sum of squares. Note that *R*^2^ calculated this way can assume negative values (when the goodness of fit for the cross-validated data is less than a zero-slope fit, i.e., the null hypothesis for regression).

To assess the significance of the prediction and to make sure that the proposed results did not reflect overfitting, we re-ran the study on a randomly permuted dataset. To do this, we shuffled the PCL-5 values (resampled without replacement), breaking the relationship between PCL-5 and fMRI data and re-ran the analysis. This process was repeated for 5000 iterations and thus quantified the ability of the model to predict noise.

Finally, the regression weights (i.e., beta coefficients) were then mapped back from PCA space to ROI-ROI functional connectivity space to identify implicated brain connections. We used the algorithm and correction proposed by Haufe et al.^39^, which provides the methodology for converting the decoding parameters into encoding parameters and make our weights interpretable (see Haufe et al.^39^ for a detailed discussion). The 1% strongest weights (50 connections) are visualized in the connectome plots. For the purpose of plotting, graphs represent the absolute value of the functional connectivity measure.

The analysis code was written in Python 3.7.1 and using scikit-learn 0.20.1 machine learning library. Connectome plots are generated using Circos 0.69–6^40^.

## Results

### Symptom scores

Our patient population had a median PCL-5 score in the moderate range, (median = PCL 46.5; 95% CI 24–71). As might be expected, PCL subscales were highly and significantly correlated with each other (Table 2, Pearson correlation coefficient, *r*, ranging between 0.38 and 0.51).

**Table 2:** Correlation between PCL-5 subscales.

	PCL-5: B	PCL-5: C	PCL-5: D	PCL-5: E	PCL-5: total
PCL-5: B	1	0.48	0.45	0.49	0.78
PCL-5: C	0.48	1	0.38	0.46	0.65
PCL-5: D	0.45	0.38	1	0.51	0.81
PCL-5: E	0.49	0.46	0.51	1	0.81
PCL-5: total	0.78	0.65	0.81	0.81	1

Criteria B, C, D, and E correspond to intrusion, avoidance, cognition/mood, and arousal/reactivity, respectively. The values in the table are Pearson’s correlation coefficients (r).

PCL-5 PTSD checklist for DSM-5.

### Machine learning output

**Fig. 1:**
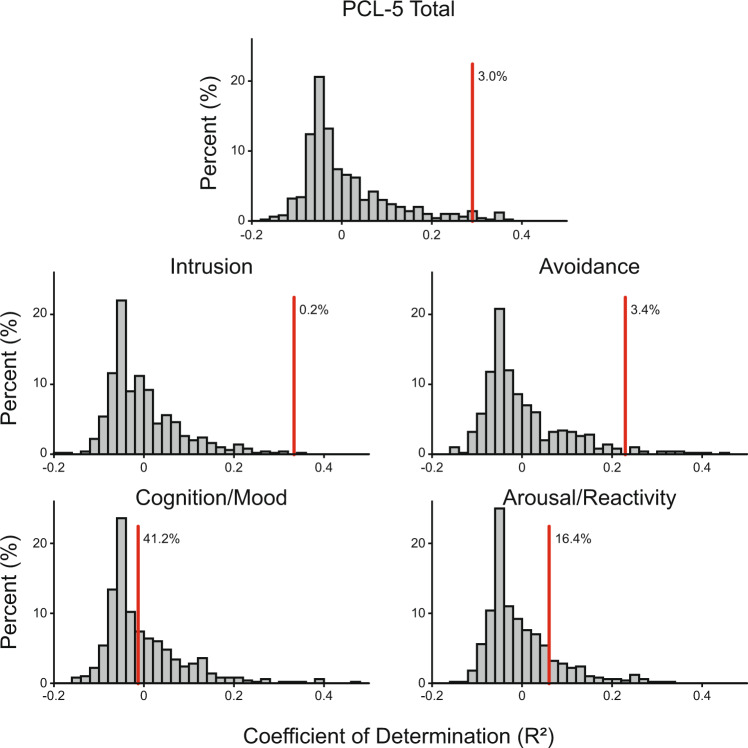
Regression performance for real and permutated data. Cross-validated regression performance (goodness of fit, *R*^2^) in predicting PCL-5 scores. The red line depicts the *R*^2^, and the histogram depicts the model’s performance in predicting shuffled data (random permutation). The percent reported next to the line shows the likelihood that the model performance can be achieved by chance. Note the measure of *R*^2^ used here can assume negative values in a leave-one-out cross-validated sample (see ‘Methods’).

**Fig. 2:**
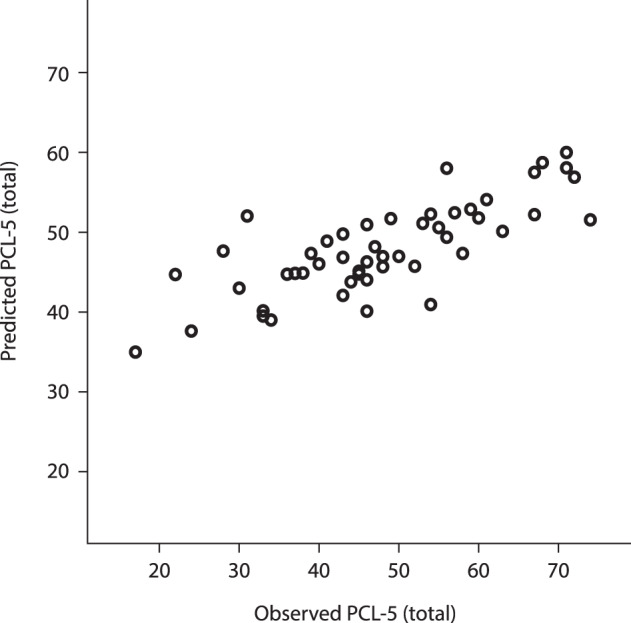
Observed vs. predicted PTSD symptom severity. The figure shown observed PCL-5 score (total PTSD symptom severity) plotted against the predicted PCL-5 score. The predictions are made on a leave-one-out cross-validated sample.

Our machine learning algorithm, utilizing feature selection and LARS regression, was able to predict total PCL-5 score as well as the subscale scores for the intrusion and avoidance clusters (quantified via coefficient of determination, *R*^2^). The *R*^2^ value for total PCL-5 was 0.29. *R*^2^ values for intrusion, avoidance, cognition, and arousal domains were 0.33, 0.23, −0.01, and 0.06, respectively. The models performed significantly better than chance in predicting total PCL-5 score (*p* = 0.030) as well as intrusion and avoidance subscales (*p* = 0.002 and *p* = 0.034). It was not able to predict cognition and arousal scores (*p* = 0.412 0.164, see Fig. 1). Model performance is presented in Fig. 2. The results were robust to leave-one-out cross-validation.

### Functional connectivity relationships predictive of symptom profiles

**Fig. 3:**
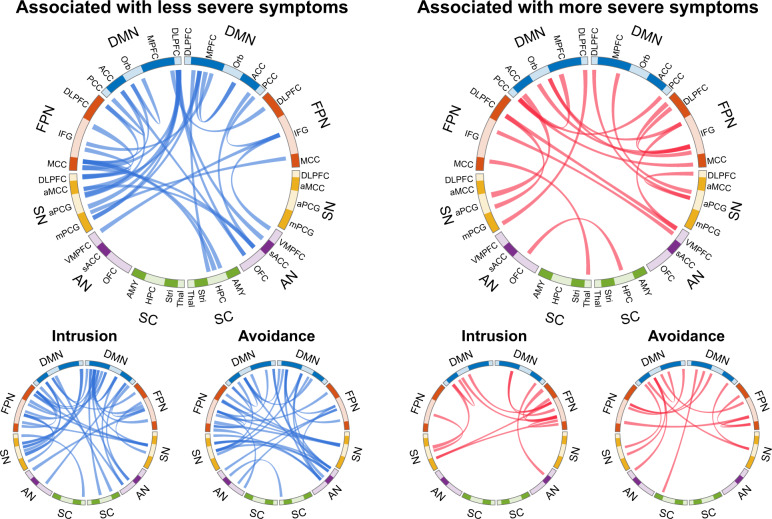
Connectivity correlates of PTSD symptom severity. Regression weights (Beta coefficients) were mapped onto functional connectivity data, and the 50 with the highest weights are plotted. Blue (plotted on the right) indicates the connections with a negative coefficient and thus associated with a lower PTSD symptoms severity, and red (plotted on the left) indicating connections with a positive coefficient and therefore associated with a more severe PTSD symptom severity. We have presented the weights predicting total PCL-5 score on top and those associated with PTSD sub-scores on the bottom. Note that the weights represent the absolute functional connectivity. DMN default mode network; DLPFC dorsolateral prefrontal cortex; MPFC medial prefrontal cortex; Orb Orbital Cortex; ACC anterior cingulate cortex; PCC posterior cingulate cortex; FPN frontoparietal network; IFG inferior frontal gyrus; MCC middle cingulate cortex; SN salience network; aMCC anterior midcingulate cortex; aPCG anterior postcingulate gyrus; mPCG medial postcingulate gyrus; AN affective network; VMPFC ventromedial prefrontal cortex; sACC subgenual anterior cingulate cortex; OFC orbitofrontal cortex; SC subcortical; AMY amygdala; HPC hippocampus; Stri striatum; Thal thalamus.

For total PCL-5, intrusion, and avoidance symptoms, we plotted the 50 connections (out of 4950) with the highest predictive weights (Fig. 3). Several connectivity patterns were predictive of PTSD symptom profiles. Increased positive connectivity between right rostral anterior cingulate (Human connectome atlas region a24; DMN) and left pars orbitalis (Human connectome region 47 l; FPN) was associated with higher intrusion and overall PCL score. Weaker DMN-to-AN functional connectivity was also observed in patients with higher scores on the same two scales. Lower positive connectivity within DMN was a feature of both intrusion and avoidance symptoms. Cross-network connectivity between DMN, SN, and FPN was also disrupted in participants with more severe intrusion and avoidance symptoms, though the direction of these effects (positive vs. negative functional connectivity) was inconsistent.

Two primary characteristics distinguished intrusion from avoidance profiles: weak within-DMN connectivity was more prominent in the high intrusion profile, while stronger within-FPN connectivity was associated with more severe symptoms of avoidance. Lower SN-to-FPN connectivity was also characteristic of high avoidance patients. Disrupted FPN-to-DMN connectivity was a feature of both intrusion and avoidance, though pairs of affected ROIs differed between profiles.

## Discussion

In this study, we adopted a novel two-step machine learning algorithm that was able to predict individual-level self-reported PTSD symptoms from resting-state functional connectivity. Our results linked severity in different PTSD symptom domains to distinct patterns of network function. This is an important first step toward defining biological diagnostic criteria for PTSD. There is an immediate need for innovations including biological markers to address the challenges that symptom heterogeneity poses for PTSD diagnosis (e.g.,^41^). Our algorithm employs a supervised learning approach that builds upon prior clinical observations enabling the identification of clinically meaningful biological patterns in a modestly sized dataset.

As technological advances have enabled the collection of more complex, larger neurobiological datasets, the use of data science techniques for high-dimensional data analysis in mental health research has gained popularity (e.g., see Refs. ^42^ and^23^). These studies primarily used ‘unsupervised’ machine learning approaches to find patterns in complex data without feedback from an outside/clinical examiner. Unfortunately, data complexity negatively impacts the performance of statistical and machine learning tools, a short-coming typically addressed by using a large number of subjects. Thus, though unsupervised techniques can be powerful, sample size requirements and associated costs limit their feasibility in clinical populations.

As we designed our machine learning algorithm, we sought to balance the challenges of analyzing high-dimensional, complex, datasets with the practical considerations of clinical research. We adopted an approach used previously in visual processing and face recognition research. Visual image data are comprised of many pixels and is thus, high dimensional. To improve model performance, machine learning studies of visual processing frequently employ a dimensionality reduction transformation (e.g., PCA) before model training^43,44^. Analogously, we applied PCA to our ROI matrix of connectivity values before regularized regression, allowing us to leverage the power of machine learning in a dataset typical of most clinical studies. Alongside this, we also limited the number of areas that were included in our analysis. We selected 100 ROIs out of a possible 376. Doing this, we deviated from a pure ‘data-driven’ approach, yet this significantly reduced the number of our features (more than 14-fold reduction, 4950 vs. 70,500) and made the analysis computationally tractable using our sample size.

The results of our analysis indicated that different symptom classes mapped onto distinct cortical networks. The model is successful in predicting the overall symptom severity, intrusion, and avoidance symptoms and associated with unique variations in functional connectivity profiles. While lower functional connectivity between DMN regions in ventrolateral PFC and affective regions in orbitofrontal cortex were common to all three of these outcomes, intrusion and avoidance symptoms were distinguished by weak within-DMN and stronger within-FPN connectivity, respectively. Although these observations await replication, the face validity between these networks and symptoms is informative given the association of DMN with self-reflection and rumination^45^ and the role of FPN in cognitive control^46^. Though stronger cognitive control may initially seem incongruent in the context of psychiatric disorder, avoidance symptoms of PTSD may represent a maladaptive control strategy. This would be consistent with other areas of research, indicating maladaptive application of cognitive control as a potential risk factor for suicide in individuals with depression^47,48^ and PTSD^49^.

While these results provide initial support for the use of biologically grounded data-driven diagnostic methods, we acknowledge that the model was unsuccessful predictions for arousal and cognition/mood symptoms. Low performance might be an artifact of the algorithm optimizing the detection of some symptoms at the expense of others, it could be due to our modest sample size, or could be driven by differences in symptom representation in our sample. Depression comorbidity^34,49^ also complicates PTSD-specific symptom classifications but reflects real-world patient symptom profiles. Another interpretation is that cognition/mood and arousal symptoms may be a product of widely distributed changes in brain function rather than prominent shifts in function in a few discrete networks. For example, if disruptions in SN related to hypervigilance are a general core feature of PTSD^50–52^ pathological threat monitoring may evoke changes in both primary sensory and associative control networks equally precluding the identification of a distinct bio-signature of arousal symptoms. Disambiguating between these alternatives requires further research in larger samples an explicit testing of comorbidity effects.

The principal limitations of this work are those inherent to secondary neuroimaging analyses using a modest sample size, cross-sectional design, and the absence of healthy controls. Because of the sample size, it is possible the results presented here reflect overfitting, in which the algorithm learns trends in the data that are not generalizable to other samples^53,54^. We attempted to mitigate this issue by using a small number of a priori defined ROIs, and implementing a data processing stream that incorporated dimensionality reduction, regularization (limiting model complexity), cross-validation, and permutation testing. Our sample was also unique in that they were largely enrolled for brain stimulation treatment studies, and thus may not represent patients with PTSD in general, and nearly all (86%) were on concurrent pharmacotherapy. Furthermore, our study did not track patients’ transition from healthy state to PTSD and as such, the results presented might be associated with the predisposition to these PTSD symptom clusters and not the symptoms themselves. Nonetheless, this study sets a precedent and introduces a methodology that can be further investigated.

In summary, we successfully generated individual-level connectivity patterns representative of self-reported PTSD symptoms. If these results can be prospectively replicated, our approach can inform the creation of individualized and objective brain-based, self-reported PTSD symptoms. Expansion of this work may yield new insights into the clinical heterogeneity of this disorder and potentially lead to individually targeted interventions for symptom reduction.

## Supplementary information

Supplementary Information
