# Supplementary material for: Mapping PTSD symptoms to brain networks: a machine learning study
Source: Transl Psychiatry. 2020 Jun 18;10:195. doi: 10.1038/s41398-020-00879-2 (PMC7303205; doi:10.1038/s41398-020-00879-2)

Supplementary Methods:

Inclusion/exclusion criteria

Participants were adults aged 18-75 years who met DSM-IV or DMS-5 criteria for PTSD; diagnoses were confirmed through interviews conducted by psychiatrists with experience in PTSD (NSP, LLC). Additional inclusion criteria were: stable doses of psychiatric medications for six weeks before study entry, ability to independently read and understand study measures and instructions. Participants were excluded for current pregnancy, breastfeeding, or plans to become pregnant within the next three months, inability to undergo magnetic resonance imaging (MRI); lifetime history of significant head injury (i.e., injury resulting in loss of consciousness greater than 10 minutes or documented evidence of resultant brain injury); current or past significant neurological disorder (e.g., seizure disorder, primary or secondary tumors in central nervous system, stroke, cerebral aneurysm); current psychotic, bipolar I disorders, alcohol and/or substance dependence disorders; active suicidal intent or plan.

The data used for the secondary analyses presented here were recorded as part of pretreatment (i.e., baseline) scanning in three previous studies from our group [1-3]. The requirement of those studies dictated some of the above criteria. 33 of 50 participants had comorbid Major Depressive disorder and were enrolled in a comorbid PTSD and MDD treatment study[1].

MRI data collection and preprocessing

Structural and functional MRI data were collected at the Brown University MRI Research Facility on either a Siemens 3T MRI scanner (Erlangen, Germany): TimTrio (n=13) or Prisma (n=37) scanner models, using a 32-channel head coil. A structural T1-weighted image was collected from each participant (TR = 1,900 ms, TE = 2.98 ms, FOV = 2562 mm, 160 slices, and voxel size = 1.0 mm isotropic). Following each structural scan, functional data was collected collected from each participant using a T2*-weighted echo-planar imaging (EPI) sequence sensitive to blood oxygenation level-dependent (BOLD) contrast (TR = 2,500ms, TE = 28ms, FOV = 1922 mm, 42 slices,

voxel size = 3.0mm isotropic, and 192 volumes). A small mirror was attached to the head coil to enable viewing of images presented on an MRI-compatible screen at the back of the scanner bore while in the MRI. Participants were instructed to passively fixate on a white crosshair presented on a black foreground while staying as still as possible during scan collection.

The CONN Toolbox[4] was used for standard and resting state-specific preprocessing steps. Standard fMRI preprocessing steps included: slice-time correction, realignment to the mean functional image, and spatial normalization to Montreal Neurological Institute (MNI) Atlas space. Structural MRI data underwent segmentation by tissue and normalization to MNI space. Preprocessing of fMRI for functional connectivity included nuisance regression of: 1) non-neuronal signal from cerebrospinal fluid and white matter per the anatomical CompCor method[5], 2) volumes that artifact detection analyses (Artifact Detection Toolbox within CONN[4]) identified as high-motion (translational>0.5, rotational>.005) or having high global signal variance (>3SD), and 3) six motion parameters estimated during realignment and their first temporal derivatives. The resulting residuals were then bandpass filtered (0.008>0.1) after subject-level nuisance regressions.

Supplementary Figure captions:

Suppl figure 1. ROI locations. ROIs based upon a subset of regions involved in reward from the high-dimensional atlas Human Connectome Project Multimodal Atlas[6]. Surface-space ROIs were converted into volumetric Montreal Neurologic Institute Atlas space with FreeSurfer[7]. Only a subset of ROIs are rendered. The prefix ‘a’ or ‘p’ typically denotes an anterior or posterior subregion, but sometimes this adjective is valid only within a smaller parcellation of a larger unimodally-defined subregion. The same is true for ‘d’, ’v’, ’r’, ’m’, ’l’, which typically stand for ‘dorsal’, ’ventral’, ‘rostral’, ’medial’, and ‘lateral’, respectively. Affective regions: 25, OFC, pOFC, 11i, 13l. Default: 10d, 10r, 10pp, a10p, p10p, 9m, d23ab, v23ab. Frontoparietal regions: 47l, a47r, 44, 45, IFSa, IFSp. 9p, a9-46v, p9-46v, 46. Salience regions: a24pr, p24pr, a32pr, 8. p32pr, 23c, 24dv, 24dd.

Suppl figure 2. Scree Plot. The plot shows the percent of data variance accounted for by each Eigen-connectome. Note that when calculating PCA, the maximum number of dimensions with non-zero Eigenvalues is the number of observations-1. As we had 50 patients, we were only able to have 49 non-zero Eigenvalues hence the X-axis goes from 1-49.

Supplementary table 1. Participant Demographics

**Age**	
Average (Standard deviation)	48.96 (11.44)
**Sex**	
M/F	33/17
**Race**	
White	84%
Black	4%
Multiracial	4%
No response	8%
**Ethnicity**	
Non-Hispanic	70%
Hispanic	2%
No response	28%
**Education**	
Less than high school	6%
High school or equivalent	14%
Some college or trade school	44%
Bachelor's degree	24%
Advanced degree	6%
No response	6%
**PCL-5 score**	
Total PCL-5	47.96 (13.58)
PCL-5, Subscale B	10.76 (4.67)
PCL-5, Subscale C	5.68 (2.42)
PCL-5, Subscale D	17.46 (6.48)

Statistics for age, total PCL-5, and PCL-5 subscales are means and standard deviations.

No response refers to the percentage of participants in which there is no response for those specific demographic questions recorded.

Education refers to the highest level of education completed. Sex is raw count. Race, ethnicity, and education is percentage of sample (n=50).

Supplementary References

1. Carpenter LL, Conelea C, Tyrka AR, Welch ES, Greenberg BD, Price LH *et al.* 5Hz Repetitive transcranial magnetic stimulation for posttraumatic stress disorder comorbid with major depressive disorder. *J Affect Disord* 2018; **235:** 414-420.

2. van 't Wout-Frank M, Shea MT, Larson VC, Greenberg BD, Philip NS. Combined transcranial direct current stimulation with virtual reality exposure for posttraumatic stress disorder: Feasibility and pilot results. *Brain Stimul* 2019; **12**(1)**:** 41-43.

3. Philip NS, Barredo J, Aiken E, Larson V, Jones RN, Shea MT *et al.* Theta-Burst Transcranial Magnetic Stimulation for Posttraumatic Stress Disorder. *Am J Psychiatry* 2019**:** appiajp201918101160.

4. Whitfield-Gabrieli S, Nieto-Castanon A. Conn: a functional connectivity toolbox for correlated and anticorrelated brain networks. *Brain Connect* 2012; **2**(3)**:** 125-141.

5. Behzadi Y, Restom K, Liau J, Liu TT. A component based noise correction method (CompCor) for BOLD and perfusion based fMRI. *Neuroimage* 2007; **37**(1)**:** 90-101.

6. Glasser MF, Coalson TS, Robinson EC, Hacker CD, Harwell J, Yacoub E *et al.* A multi-modal parcellation of human cerebral cortex. *Nature* 2016; **536**(7615)**:** 171-178.

7. Fischl B. FreeSurfer. *Neuroimage* 2012; **62**(2)**:** 774-781.

Supplementary Figure 1: Cortical and subcortical regions used in the analysis

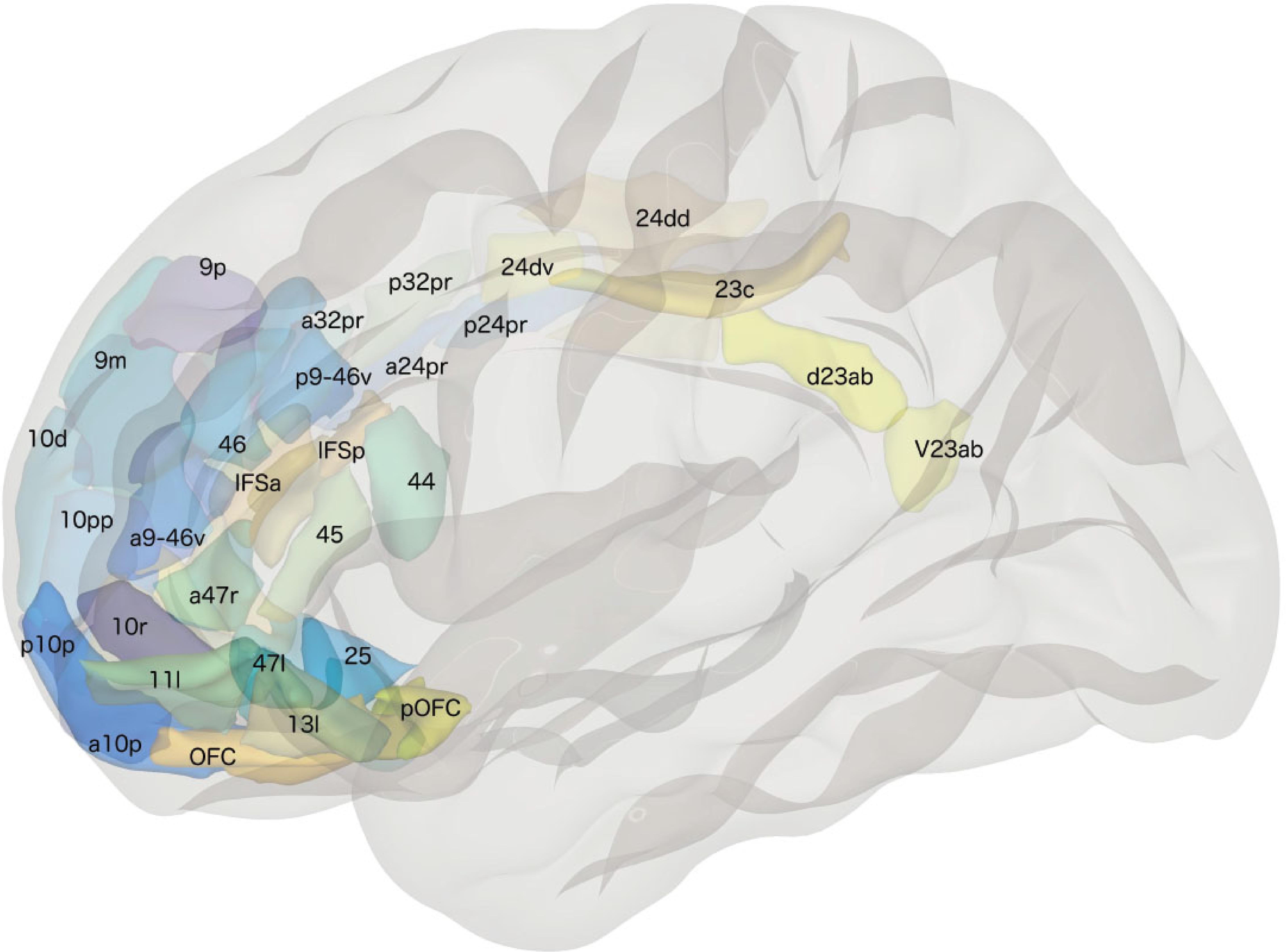

Supplementary Figure 2: Scree plot showing the proportion of variance described by different eigen-connectomes

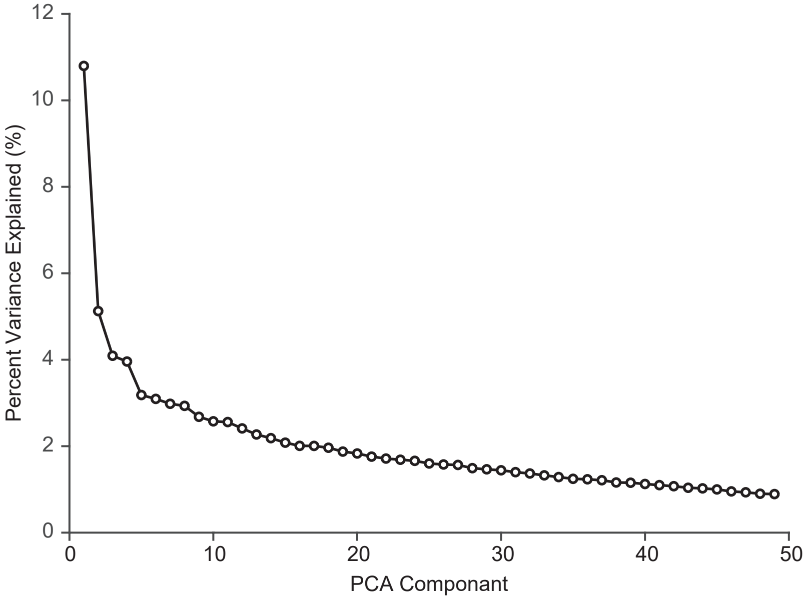

Supplement: Supplementary file 1 — Supplementary Information [file 41398_2020_879_MOESM1_ESM.pdf]
